# Prevalence, influencing factors, and dementia outcome of sarcopenic obesity in China

**DOI:** 10.1007/s40520-025-03318-8

**Published:** 2026-01-13

**Authors:** Xianzhi Li, Yajie Li, Meiying Shen, Zonglei Zhou, Shunjin Liu, Li Yin, Qian Zhu, Xiangyi Xing

**Affiliations:** 1 Meteorological Medical Research Center, Panzhihua Central Hospital, Panzhihua, China; 2 Clinical Medical Research Center, Panzhihua Central Hospital, Panzhihua, China; 3 Tibet Center for Disease Control and Prevention, Lhasa, China; 4 Nursing Department, Panzhihua Central Hospital, Panzhihua, China; 5 Shanghai Pudong Hospital, Fudan University Pudong Medical Center, Shanghai, China; 6 Department of Pharmacy, Panzhihua Central Hospital, Panzhihua, China; 7 No.34, Yikang Street, East District, Panzhihua, 617067 China

**Keywords:** Sarcopenic obesity, Prevalence, Influencing factors, Geographic disparities, Dementia

## Abstract

**Background:**

This study examines the epidemiology of sarcopenic obesity (SO) in China, focusing on national prevalence, modifiable influencing factors, and its longitudinal association with dementia risk in middle-aged and older adults.

**Methods:**

Using the 2015 wave of the China Health and Retirement Longitudinal Study (CHARLS), SO prevalence was estimated nationally and regionally using Bayesian spatial modeling. Modifiable influencing factors were identified via multivariable logistic regression, with their relative contributions quantified using Extreme Gradient Boosting (XGBoost). The association between baseline SO (2015) and incident dementia risk (2018) was assessed using multivariable logistic regression.

**Results:**

Among 10,256 participants aged ≥ 45 years, the national SO prevalence was 6.40% (95% Bayesian confidence intervals [BCIs]: 6.39–6.41%), with significant regional variation (Northern region: 8.60% [8.59–8.61%]; Southern region: 4.90% [4.86–4.94%]). Older age, female sex, hypertension, and depressive symptoms showed statistically significant associations with SO. Age emerged as the strongest predictor in XGBoost analysis. Compared to normal-weight individuals without sarcopenia, those with normal-weight sarcopenia had 63% higher dementia risk (odds ratio [OR] = 1.63; 95% confidence interval [CI]: 1.04–2.58), while SO individuals showed 89% increased risk (OR = 1.89; 95% CI: 1.67–2.15).

**Conclusion:**

These findings emphasize the significant geographic disparity in SO prevalence across China and reveal that SO is associated with a substantially elevated risk of dementia, underscoring the need for targeted interventions in aging populations.

**Supplementary Information:**

The online version contains supplementary material available at 10.1007/s40520-025-03318-8.

## Introduction

Sarcopenic obesity (SO) is a clinical condition characterized by the coexistence of sarcopenia (an age-related decline in muscle mass and function) and obesity (marked by excessive adipose tissue accumulation) [1]. Recognized as a growing public health concern, SO poses a significant threat to middle-aged and elderly populations [2]. Some researchers argue that SO is not merely a combination of two pathological conditions but rather a synergistic interaction that exacerbates metabolic and functional impairments [3]. This condition increases the risk of disability, cardiovascular and metabolic diseases, and mortality [4, 5]. Given that the medical consequences of SO are more severe than those of sarcopenia or obesity alone, SO is regarded as a critical public health challenge in aging societies [2].

The prevalence of SO is rising, particularly among aging populations, with global estimates suggesting that more than 10% of older adults may be affected [6–8]. Reported prevalence rates vary widely, ranging from 2.75% to over 20%, depending on diagnostic criteria and body composition assessment methods. In mainland China, several studies have estimated SO prevalence, but findings exhibit considerable heterogeneity [9–13]. For instance, Du et al. (2019) reported a prevalence of 4.0% in a community-based sample of 213 men and 418 women aged ≥ 65 years in East China [9], whereas Ma et al. (2020) found a rate of 9.2% among 303 suburban residents in Tianjin [10]. Similarly, Meng et al. (2014) observed an 11.5% prevalence in 101 men aged ≥ 80 years in Beijing [11], while Wang et al. (2019) and Yang et al. (2015) reported rates of 6.0% and 7.2%, respectively, in larger community-dwelling populations [12, 13]. However, these studies are limited by small sample sizes, restricted geographic coverage, and demographic constraints (e.g., specific age or gender groups), hindering a comprehensive assessment of SO’s overall and region-specific prevalence in mainland China. Consequently, a nationwide, large-scale epidemiological study is warranted to accurately determine SO’s disease burden and inform evidence-based healthcare resource allocation.

The current understanding of SO remains limited due to its complex pathogenesis and poorly characterized molecular mechanisms, which has impeded the development of effective prevention and treatment strategies [14]. Advancing etiological research to identify key risk factors is therefore essential for establishing evidence-based interventions to address this growing public health concern. However, potential influencing factors contributing to SO development in Chinese populations remain poorly understood.

Dementia is an umbrella term encompassing various brain disorders characterized by cognitive impairment affecting memory, thinking, and emotional regulation. Globally, dementia cases are projected to increase dramatically from 57 million in 2019 to 152 million by 2050, primarily affecting aging populations [15]. While the exact etiology remains unclear [16], multiple risk factors have been identified, including advanced age, chronic diseases, and genetic predisposition [16]. The relationship between obesity and dementia remains controversial: while some studies identify late-life obesity as an independent risk factor, others report an inverse association - the so-called “obesity paradox” [17]. Similarly, sarcopenia has emerged as a potential dementia risk factor [18]. SO, which may confer greater health risks than either condition alone (sarcopenia or obesity) [19], has been associated with cognitive impairment in several observational studies [20, 21]. However, evidence linking SO directly to dementia remains scarce. To date, only three relevant studies were identified in the existing literature [16, 20, 22]. Among these, two employed a cross-sectional design [20, 22], which inherently limits causal inference. Notably, the study by Someya et al. [20] had a relatively small sample size, further restricting the generalizability of its findings. The sole prospective evidence currently available stems from a UK Biobank cohort study [16], which systematically examined the association between sarcopenic obesity and dementia. However, given the substantial differences between Asian and European populations in body composition, lifestyle, genetic susceptibility, and the rising prevalence of sarcopenic obesity among older adults in China [23], a prospective study in a Chinese population is therefore necessary.

To address these research gaps, we utilized data from the China Health and Retirement Longitudinal Study (CHARLS) to: [1] estimate the national and regional prevalence of SO; [2] identify potential influencing factors associated with SO development; and [3] examine the associations between SO and dementia risk among middle-aged and older Chinese adults. The findings will provide critical evidence to inform SO prevention strategies and establishes a foundation for future research on aging-related health outcomes in China.

## Methods

### Data collection and study population

This study draws data from CHARLS, whose study design and participant details have been previously published [24]. Briefly, CHARLS used multistage probability sampling to gather comprehensive health data from 450 communities across 28 provinces in mainland China. Launched in 2011, the study initially enrolled over 17,000 participants from approximately 10,000 households, with follow-ups conducted every 2–3 years through 2020. The dataset includes eight core modules covering demographic characteristics, health status, and biometric measurements, offering a robust basis for examining SO epidemiology in middle-aged and older adults. The study protocol received ethical approval from Peking University’s ethics review committee (IRB00001052-11015), and all participants provided written informed consent.

The current study comprised two distinct analytical components:

1) Prevalence and influencing factor analysis

Utilizing data from the 2015 follow-up (wave 3), we examined SO prevalence and influencing factors. Exclusion criteria included: incomplete sarcopenia or waist circumference (WC) measurements, age < 45 years, or missing covariate data. The final analysis included 10,256 participants (Figure S1). Mainland China’s provinces and municipalities were classified into seven geographic regions according to the national administrative divisions: Northeast, North, Central, East, South, Northwest, and Southwest (Table S1) [25].

2) SO-Dementia association analysis

This section employed a longitudinal design, utilizing wave 3 (2015) data as baseline and wave 4 (2018) data as follow-up. We excluded participants who lacked dementia assessments in either wave or had a dementia diagnosis at baseline (*N* = 4,558; Figure S1). This study design significantly strengthens the capacity for causal inference regarding the SO-dementia association.

### Ascertainment of SO status

SO was defined as the co-occurrence of obesity and sarcopenia. Obesity was determined based on WC, using sex-specific thresholds of > 85 cm for men and > 80 cm for women [26]. Sarcopenia was identified according to a modified version of the 2019 AWGS criteria, adapted for the CHARLS dataset, which lacks direct appendicular skeletal muscle mass (ASM) measurements by dual-energy X-ray absorptiometry (DXA) or bioelectrical impedance analysis (BIA) [27, 28]. Specifically, participants were classified as having sarcopenia if they presented with low muscle mass, plus either low muscle strength or poor physical performance. Low muscle strength was defined as handgrip strength < 28 kg for men and < 18 kg for women. Poor physical performance was indicated by a time ≥ 12 seconds on the five-time chair stand test. For low muscle mass, we applied sex-specific cut-offs for the appendicular skeletal muscle mass index (ASMI) derived from prior external validation studies (≤ 7.00 kg/m² for men and ≤ 5.28 kg/m² for women), as direct ASM measurement was unavailable in CHARLS [27, 28]. Detailed measurement protocols for all indicators are described in the ‘Definition and Ascertainment of Sarcopenia’ section of the supplementary materials.

### Assessment of dementia

This study defines dementia as the concurrent presence of functional impairment and cognitive impairment or a self-reported physician diagnosis of dementia. This definition aligns with the diagnostic criteria for dementia outlined in the Diagnostic and Statistical Manual of Mental Disorders (Fifth Edition) (DSM-5) and the International Classification of Diseases (Tenth Edition) (ICD-10), and its validity has been corroborated by prior research [29]. For participants without cognitive score data, dementia cases were identified based on an affirmative response to the question: “Have you been diagnosed with dementia by a doctor?” [24, 30].

Functional impairment was assessed using the Katz Activities of Daily Living scale (ADLs) in CHARLS [31], which evaluates six basic ADLs: bathing, transferring (getting in and out of bed), dressing, toileting, eating, and continence (controlling urination and defecation). Participants were classified as having functional impairment if they required assistance in any ADLs.

Cognitive function was assessed using the adapted version of Telephone Interview for Cognitive Status (TICS) [24], which provides a total score of 31 points across four domains: time orientation, episodic memory, calculation ability, and figure drawing [32].

- Time orientation (5 points): Participants were asked about the current year, month, day, day of the week, and season. One point was awarded for each correct answer.
- Episodic memory (20 points): This was evaluated by immediate and delayed recall of 10 random words. The interviewer read a list of 10 words, and the number of words immediately recalled was recorded (immediate recall). After completing other assessments (including a depression scale, calculation, and drawing tests), participants were asked to recall the same words again (delayed recall). One point was given for each correctly recalled word in each trial.
- Calculation ability (5 points): Participants were asked to serially subtract 7 from 100 on five consecutive occasions. One point was awarded for each correct subtraction.
- Figure drawing (1 point): Participants were shown a picture of two overlapping pentagons and were asked to reproduce the drawing. One point was awarded for a correct reproduction.

The TICS-based global cognitive scores were calculated as the sum of the scores from the four domains, with higher scores indicating better cognitive performance. To define cognitive impairment, we adopted a threshold corresponding to the 22.24th percentile of TICS-based global cognitive scores distribution, consistent with the reported prevalence of cognitive impairment (22.24%) among Chinese older adults as measured by the MMSE [33]. This percentile corresponded to a cutoff score of 8 in our study. Consequently, participants with a global cognitive score of 8 or lower were classified as having cognitive impairment.

### Potential influencing factors

To examine influencing factors of SO, we performed multivariable logistic regression analysis. A series of potential influencing factors were included in this study, including age, gender, marital status, residence, drinking status, hypertension, diabetes, depressive status, and social participation. All of these factors were entered simultaneously into the multivariable logistic regression model. More details of these factors are shown in Table S2. Marital status was determined by the participants’ self-reports. Residence is divided into urban and rural based on the participants’ household registration. Hypertension and diabetes were defined as having been diagnosed by a medical professional. Depressive symptoms were assessed using the 10-question version of the Center for Epidemiological Studies Depression Scale (CES-D), with scores ≥ 10 indicating depressive symptoms [34]. Drinking status was classified as “never drinking”, “have drunk alcohol at present or before”, based on the frequency of alcohol consumption in the past year. Social participation was operationalized as engagement in at least one of the following activities within the preceding month [35]: interacted with friends; played Ma-jong, played chess, played cards, or went to community club; provided help to family, friends, or neighbors who do not live with you; went to a sport, social, or other kind of club; took part in a community-related organization; done voluntary or charity work; cared for a sick or disabled adult who does not live with you; attended an educational or training course; stock investment; or used the Internet.

### Statistical analysis

Categorical variables were reported as frequencies (percentages), and continuous variables were expressed as means ± standard deviations (SD). Group differences were evaluated using chi-square tests for categorical variables and analysis of variance (ANOVA) for continuous variables.

To estimate the prevalence of SO while accounting for influencing factors and potential spatial correlation of regions, we utilized a Bayesian hierarchical model. This model incorporated a conditional autoregressive (CAR) prior to account for spatial-specific random effects [36]. By directly modeling spatial dependence, this approach enables prevalence estimates for each region to be informed by data from neighboring regions, thereby improving the stability of estimates in regions with sparse data and mitigating small-area bias. More details regarding the methodology can be found in the previous literature [37]. The model was implemented in “Rjags” using Markov chain Monte Carlo (MCMC) sampling. We ran three independent chains for 5,000 iterations following a burn-in period and confirmed satisfactory convergence using Gelman-Rubin statistics (R-hat < 1.1 for all key parameters). To ensure national representativeness for the middle-aged and older population, we incorporated the complex survey design of the CHARLS data directly into the model by applying the individual sampling weights from the 2015 biomarker and physical examination module.

Potential influencing factors for SO were first examined using multivariate logistic regression, adjusting for age, sex, marital status, hypertension, diabetes, depression status, residence, alcohol consumption, and social participation, based on previous studies [38]. Multicollinearity among covariates was assessed using the variance inflation factor (VIF), with no variables exceeding the threshold of VIF > 10 [39]. Additionally, an Extreme Gradient Boosting (XGBoost) model was employed to evaluate the relative contribution of each influencing factor. This algorithm iteratively constructs an ensemble of decision trees, with each new tree built to fit the residuals from the previous ensemble. It incorporates regularization terms to control model complexity and mitigate overfitting, and efficiently handles missing or sparse data [40]. Key hyperparameters for the model were set as follows: the maximum tree depth was 14, the learning rate was 0.5, and the number of boosting rounds was 14. Variable importance was quantified using the built-in ‘gain’ metric, which represents the average information gain brought by each feature across all splits in the decision trees. Subsequently, stratified analyses by seven geographic regions were conducted to assess regional variations in influencing factor effect sizes [41].

Multivariate binary logistic regression models were also used to examine the association between SO and dementia, adjusting for age, sex, marital status, hypertension, diabetes, depression status, residence, alcohol consumption, and social participation, based on previous studies. Mixed linear model was used to estimate the relationship between SO and TICS-based global cognitive scores, adjusting for the same covariates mentioned above. Considering that TICS-based global cognitive scores were not normally distributed, we convert it by arithmetic square root to satisfy the normal assumption of the mixed linear model for the dependent variable. The model is as follows:

$$\:\sqrt{{\mathrm{T}\mathrm{I}\mathrm{C}\mathrm{S}-\mathrm{b}\mathrm{a}\mathrm{s}\mathrm{e}\mathrm{d}\:\mathrm{g}\mathrm{l}\mathrm{o}\mathrm{b}\mathrm{a}\mathrm{l}\:\mathrm{c}\mathrm{o}\mathrm{g}\mathrm{n}\mathrm{i}\mathrm{t}\mathrm{i}\mathrm{v}\mathrm{e}\:\mathrm{s}\mathrm{c}\mathrm{o}\mathrm{r}\mathrm{e}\mathrm{s}}_{i,j}}={\beta\:}_{0}+{\beta\:x}_{i,j}+{\gamma\:z}_{i,j}+{city}_{i}+{\mu\:}_{\left(i\right)}$$

where *i* denotes the subject index; *j* represents visit index; *β*_*0*_ refers to the intercept; *β* is the regression coefficients for SO; *z*_*i, j*_ denotes a set of adjusted covariates; *γ* denotes the corresponding coefficients; *city*_*i*_ represents a fixed-effect term to control the unmeasured city-specific risk factors of TICS-based global cognitive scores; and *µ* denotes a random term to model the correlations between repeated measurements from the same subject over two waves.

To address missing values in the covariates, multiple imputation was performed using the Multivariate Imputation by Chained Equations (MICE) approach, implemented with the *mice* package in R. Variables with missing data included age, sex, marital status, hypertension, depressive symptoms, drinking status, diabetes, residence, social participation, and SO status. Predictive mean matching (PMM) was used as the primary imputation method. Five independent imputed datasets were generated, with the number of iterations per dataset set to 50 to ensure convergence of the imputation algorithm, and a fixed random seed (500) was set to ensure reproducibility. In subsequent analyses, models were fitted separately on each of the five imputed datasets, and the results were pooled using Rubin’s rules to obtain combined parameter estimates (e.g., odds ratios) and standard errors for final statistical inference and reporting.

All analyses were performed using R software (version 4.2.2). A two-tailed p-value < 0.05 was considered statistically significant.

## Results

### Characteristics of the study participants

The final analytical sample included 10,256 participants aged ≥ 45 years (51.07% female), including 655 (6.39%) individuals with SO. Most participants were married (88.94%), normotensive (91.62%), free from depressive symptoms (67.86%), non-drinkers (62.42%), non-diabetic (97.77%), engaged in social activities (55.82%), and rural residents (76.31%). Significant regional variations (*P* < 0.001) were found in SO prevalence and most sociodemographic and health characteristics across China’s seven major geographic regions (Table 1).

**Table 1:** Characteristics comparison according to geographical region in survey 2015

Characteristics (2015)	Overall (*N* = 10256)	Central (*N* = 1549)	East (*N* = 3239)	North (*N* = 755)	Northeast (*N* = 1150)	Northwest (*N* = 776)	South (*N* = 920)	Southwest (*N* = 1867)	*P* value
Age (year), mean (SD)	59.20 (9.54)	58.95 (9.45)	59.52 (9.69)	59.25 (9.33)	57.78 (9.06)	57.69 (9.23)	60.10 (9.65)	59.89 (9.65)	< 0.001
Gender, n (%)									
Male	5018 (48.93)	758 (48.93)	1571 (48.50)	380 (50.33)	558 (48.52)	380 (48.97)	435 (47.28)	936 (50.13)	0.810
Female	5238 (51.07)	791 (51.07)	1668 (51.50)	375 (49.67)	592 (51.48)	396 (51.03)	485 (52.72)	931 (49.87)	
Marital status, n (%)									
Married	9122 (88.94)	1419 (91.61)	2872 (88.67)	659 (87.28)	1056 (91.83)	669 (86.21)	799 (86.85)	1648 (88.27)	< 0.001
Unmarried	1134 (11.06)	130 (8.39)	367 (11.33)	96 (12.72)	94 (8.17)	107 (13.79)	121 (13.15)	219 (11.73)	
Hypertension, n (%)									
Yes	859 (8.38)	110 (7.10)	281 (8.68)	53 (7.02)	138 (12.00)	57 (7.35)	62 (6.74)	158 (8.46)	< 0.001
No	9397 (91.62)	1439 (92.90)	2958 (91.32)	702 (92.98)	1012 (88.00)	719 (92.65)	858 (93.26)	1709 (91.54)	
Depressive symptoms, n (%)									
No	6960 (67.86)	1062 (68.56)	2317 (71.53)	537 (71.13)	818 (71.13)	447 (57.60)	653 (70.98)	1126 (60.31)	< 0.001
Yes	3296 (32.14)	487 (31.44)	922 (28.47)	218 (28.87)	332 (28.87)	329 (42.40)	267 (29.02)	741 (39.69)	
Drinking status, n (%)									
Yes	3854 (37.58)	550 (35.51)	1254 (38.72)	247 (32.72)	496 (43.13)	254 (32.73)	325 (35.33)	728 (38.99)	< 0.001
No	6402 (62.42)	999 (64.49)	1985 (61.28)	508 (67.28)	654 (56.87)	522 (67.27)	595 (64.67)	1139 (61.01)	
Diabetes, n (%)									
Yes	229 (2.23)	36 (2.32)	68 (2.10)	26 (3.44)	34 (2.96)	15 (1.93)	12 (1.30)	38 (2.04)	0.053
No	10,027 (97.77)	1513 (97.68)	3171 (97.90)	729 (96.56)	1116 (97.04)	761 (98.07)	908 (98.70)	1829 (97.96)	
Residence, n (%)									
Urban	2430 (23.69)	354 (22.85)	687 (21.21)	175 (23.18)	462 (40.17)	189 (24.36)	227 (24.67)	336 (18.00)	< 0.001
Rural	7826 (76.31)	1195 (77.15)	2552 (78.79)	580 (76.82)	688 (59.83)	587 (75.64)	693 (75.33)	1531 (82.00)	
Social participation, n (%)									
No	4531 (44.18)	603 (38.93)	1438 (44.40)	277 (36.69)	458 (39.83)	374 (48.20)	415 (45.11)	966 (51.74)	< 0.001
Yes	5725 (55.82)	946 (61.07)	1801 (55.60)	478 (63.31)	692 (60.17)	402 (51.80)	505 (54.89)	901 (48.26)	
SO, n (%)									
No	9601 (93.61)	1470 (94.90)	3045 (94.01)	690 (91.39)	1085 (94.35)	715 (92.14)	875 (95.11)	1721 (92.18)	< 0.001
Yes	655 (6.39)	79 (5.10)	194 (5.99)	65 (8.61)	65 (5.65)	61 (7.86)	45 (4.89)	146 (7.82)	

*Notes*: SD, standard deviation; SO, sarcopenia obesity

The spatial distribution of all nine covariates is presented in Figure S2. Region-specific analyses showed distinct geographical patterns: depressive symptoms were most prevalent in Northwest China (42.40%), while diabetes prevalence was highest in the North (3.44%). Alcohol consumption rates peaked in both the Northeast (43.13%) and Southwest (38.99%). Sex distribution varied regionally, with the highest male proportion in the North (50.33%) and female predominance in the South (52.72%). Hypertension was most common in the Northeast (12.00%), which also had the largest urban population concentration (40.17%). The North reported the highest social participation rate (63.31%), whereas marriage rates were greatest in the Northeast (91.83%) and Central China (91.61%) (Table 1; Figure S2).

### Prevalence of SO

Table 2 presents the estimated prevalence of SO. In 2015, the overall SO prevalence was 6.40% (95% BCI: 6.39–6.41). Regionally, the highest prevalence rates were observed in North (8.60%; 95% BCI: 8.59–8.61), Northwest (7.90%; 95% BCI: 7.89–7.91), and Southwest (7.80%; 95% BCI: 7.79–7.81) China, while the South exhibited the lowest prevalence (4.90%; 95% BCI: 4.86–4.94) (Table 2, panel A of Figure S2).

**Table 2:** Prevalence and its 95% BCI of SO globally and in seven geographical regions

Regions	Prevalence (95% BCI)
Overall	6.40% (6.39%, 6.41%)
Central	5.10% (5.09%, 5.11%)
East	6.00% (5.98%, 6.02%)
North	8.60% (8.59%, 8.61%)
Northeast	5.70% (5.67%, 5.73%)
Northwest	7.90% (7.89%, 7.91%)
South	4.90% (4.86%, 4.94%)
Southwest	7.80% (7.79%, 7.81%)

*Notes*: BCI, Bayesian confidence interval; SO, sarcopenia obesity

### Influencing factors for SO

Older age (OR = 1.09, 95% CI: 1.08–1.10), female sex (OR = 4.86; 95% CI: 3.91–6.10), hypertension (OR = 1.62; 95% CI: 1.25–2.08), and depressive symptoms (OR = 1.52; 95% CI: 1.28–1.81) showed statistically significant associations with risk of SO (Table 3). The XGBoost algorithm quantified the relative contributions of influencing factors, revealing the following order of importance: age (highest), gender, depressive symptom, and hypertension (lowest) (Figure S3).

**Table 3:** Factors associated with SO in CHARLS 2015

Risk factors	OR	*P* value
Age (per 1-year increment)	1.09 (1.08, 1.10)	< 0.001
Gender (Female vs. Male)	4.86 (3.91, 6.10)	< 0.001
Hypertension	1.62 (1.25, 2.08)	< 0.001
Depressive symptom	1.52 (1.28, 1.81)	< 0.001

*Notes*: SO, sarcopenia obesity; OR, odds ratio

**Fig. 1:**
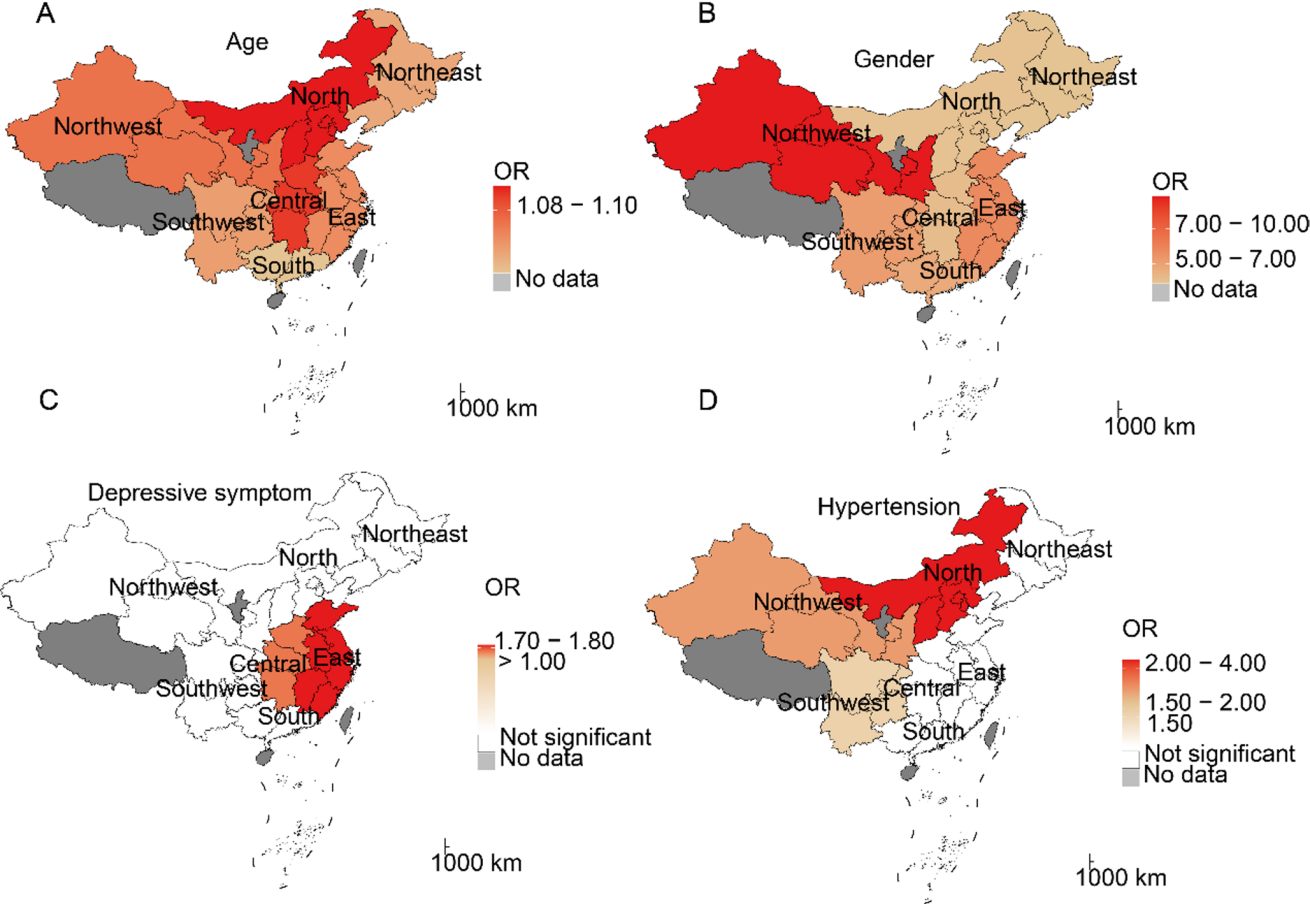
Region-specific regression coefficients of SO risk factors. *Notes*: OR, odds ratio; SO, sarcopenia obesity

The effects of influencing factors for SO showed modest variation across regions (Fig. 1). For example, increasing age was associated with a higher risk of SO. The highest effect is observed in North (OR = 1.11; 95% CI: 1.07–1.15) and Central (OR = 1.11; 95% CI: 1.08–1.14) China, while the lowest is in South China (OR = 1.08; 95% CI: 1.04–1.12). Additional details can be found in Fig. 1 and Table S3.

### Associations between SO with dementia

Among 5,698 participants, 368 (6.46%) were diagnosed with dementia. Compared to the reference group (normal weight and without sarcopenia), individuals with normal weight and sarcopenia had a 63% higher dementia risk (OR = 1.63; 95% CI: 1.04–2.58), while those with SO had an 89% higher risk (OR = 1.89; 95% CI: 1.67–2.15) (*P* for trend < 0.001). Both groups also demonstrated significantly lower TICS-based global cognitive scores (*P* for trend < 0.001) (Table 4).

**Table 4:** Associations between SO with dementia and TICS-based global cognitive scores

Outcome	Effect values	-
Dementia	OR (95% CI)	*P* value
Group 1	1.00 (Reference)	-
Group 2	0.97 (0.81, 1.17)	0.770
Group 3	1.63 (1.04, 2.58)	< 0.001
Group 4	1.89 (1.67, 2.15)	0.030
*P* for trend	< 0.001	-
TICS-based global cognitive scores	β (95% CI)	P value
Group 1	1.00 (Reference)	-
Group 2	0.12 (−0.42, 0.66)	0.660
Group 3	−2.23 (−3.57, −0.89)	< 0.001
Group 4	−2.26 (−2.63, −1.88)	0.001
*P* for trend	< 0.001	-

*Notes*: SO, obesity sarcopenia; TICS, Telephone Interview for Cognitive Status; OR, odds ratio; CI, confidence interval; β, beta; model was adjusted for age, gender, marital status, hypertension, diabetes, depression status, residence, alcohol consumption, and social participation. Group 1: normal weight and without sarcopenia; Group 2: obesity and without sarcopenia; Group 3: normal weight and with sarcopenia; Group 4: SO

### Sensitivity analysis

To assess the robustness and generalizability of our findings, we performed sensitivity analyses comparing results from complete-case datasets with those obtained after multiple imputation. These analyses (Figure S4, Table S4) support both the stability of our findings and the reliability of the imputed data.

## Discussion

This large-scale, nationally representative study highlights several important findings. First, the overall prevalence of SO in 2015 was 6.40% (95% BCI: 6.39%, 6.41%), with significant regional disparities across China. The highest prevalence rates were found in the North, Northwest, and Southwest regions, while the South exhibited the lowest rates. Second, several independent influencing factors for SO were identified, including older age, female sex, hypertension, and more severe depressive symptoms—with age demonstrating the highest relative contribution. Third, individuals with SO, as well as those with normal weight but sarcopenia alone, had significantly higher odds of dementia compared to individuals with normal weight and no sarcopenia.

The overall prevalence of SO in our study was 6.40%, which is notably lower than the previously reported rates of 14% [42] and 11% [6]. This discrepancy may be attributed to the relatively younger age of our study population compared to those in prior studies. The global prevalence of SO remains poorly characterized due to the lack of standardized diagnostic criteria worldwide [43]. This inconsistency in definitions and assessment methods has resulted in limited epidemiological data and substantial variations in reported prevalence rates across different ethnicities and age groups. Current evidence suggests that the worldwide prevalence of SO ranges from 5% to 10%, with significant variations observed across ethnic groups and geographic regions [44]. These differences likely reflect both true population variations and methodological inconsistencies in case identification.

Our findings reveal a distinct north-south geographic gradient in SO prevalence, with significantly higher rates observed in northern regions compared to their southern counterparts. This spatial pattern may be attributed to several potential factors: [1] documented regional differences in lifestyle factors such as dietary patterns, physical activity levels and alcohol drinking [45, 46]; [2] variations in climate conditions that may influence energy expenditure and body composition [37, 47]; [3] potential genetic or epigenetic variations across populations; and [4] the relatively higher risk impacts of hypertension and depressive symptoms in north China compared to the south [37, 48]. The consistency of this geographic pattern across our study population suggests the possible influence of environmental or socioeconomic determinants that warrant further investigation in future studies. In addition, significant regional variation was observed in the impact of influencing factors, with the highest risk effects for age, gender, depressive symptoms, and hypertension occurring in the North, Northwest, East, and North, respectively. Geographical variation in SO prevalence might be partially explained by the higher risk impacts of age and hypertension in northern China compared to the south, as revealed by our analysis. This novel evidence suggests that northern provinces warrant prioritized interventions for SO control.

Consistent with prior evidence [49, 50], our findings confirmed that female sex and older age were independently associated with SO, with age emerging as the most significant contributing factor. This elevated risk may stem from age-related muscle loss, hormonal changes, and metabolic alterations that promote fat accumulation alongside reduced lean mass [38]. Aging can affect body composition, especially by altering the distribution of muscles and fat, often accompanied by a gradual decrease in muscle mass and an increase in fat mass [51]. Muscle mass undergoes developmental increases during adolescence, reaches its maximum between 30 and 40 years of age, and subsequently declines rapidly after the seventh decade of life. This age-related musculoskeletal deterioration is accompanied by progressive reductions in resting metabolic rate, thermic effect of food, and physical activity levels, collectively resulting in diminished total energy expenditure. These metabolic changes drive a gradual accumulation of body fat that typically peaks around age 70 before entering a decline phase. The postmenopausal decline in estrogen and other sex hormones removes their protective effects on musculoskeletal and metabolic systems [52, 53], leading to impaired muscle protein synthesis via downregulation of anabolic pathways and promoting visceral adiposity through adipokine dysregulation. Besides, women’s intrinsically lower baseline skeletal muscle mass renders them disproportionately susceptible to the functional consequences of age-related lean tissue depletion.

Our study found that hypertension and depressive symptoms were also influencing factors for SO, while hypertension had a relatively small impact on SO. This finding aligns with existing literature and expert consensus assessments [38], where hypertension was frequently reported in association with SO but consistently rated as having lower clinical relevance compared to other metabolic and psychological risk factors. While the association between depression and obesity is well-established [54], the relationship with SO remains less clear. A recent systematic review examining depressive disorders and SO revealed significant methodological heterogeneity in assessment approaches, contributing to inconsistent findings across studies [55].

Several studies have explored the association between SO and cognitive function in middle-aged and older adults, consistently finding that SO is linked to cognitive decline [12, 21]. However, to date, only one study has investigated the longitudinal relationship between SO and dementia [16]. This longitudinal study utilized UK Biobank data, including 208,867 participants aged 60–69 at baseline, to examine the associations of obesity, sarcopenia, and SO with dementia risk. The findings demonstrated that SO was significantly associated with an increased risk of dementia and earlier onset of the disease [16]. Although this prior study shares a prospective cohort design with ours, there are notable differences in the diagnostic criteria for sarcopenia and obesity. Despite these methodological variations, the overall conclusions align with our findings. Unlike the EWGSOP 2018 guidelines [56], which emphasize muscle strength, the AWGS 2019 criteria [57] consider both muscle strength and physical performance equally important in defining sarcopenia. Given the significant differences between Asian and Western populations in body size, composition, and physical activity patterns, our study adopted the AWGS 2019 standards, which are more suitable for Asian populations, to diagnose SO. Notably, our findings demonstrate that individuals with SO face the greatest dementia risk, followed by those with sarcopenia alone, suggesting a synergistic interaction between muscle loss and obesity in cognitive decline. This phenomenon may be explained by the amplified pro-inflammatory state resulting from the pathological crosstalk between adipokines and myokines, which appears to exert more detrimental neurological effects than either metabolic dysfunction or muscle atrophy alone [58]. The compounding impact of these interacting pathways underscores the critical need for interventions that simultaneously address both sarcopenia and obesity in dementia prevention strategies.

Emerging evidence suggests that sarcopenic obesity potentiates dementia risk through three interrelated pathological pathways: [1] the dualistic adipokine imbalance where obesity concurrently enhances neuroprotective factor secretion and elevates synapse-damaging pro-inflammatory cytokines like IL-6, creating complex neurological consequences [59]; [2] the disrupted myokine signaling wherein muscle-derived factors such as irisin dysregulate critical cognitive processes including neuroinflammatory control, astrocyte function, and hippocampal neurotrophin production [60]; and [3] the maladaptive adipose-muscle axis where pathological cross-talk between these tissues amplifies systemic inflammation as demonstrated in experimental models [58]. Particularly significant is obesity’s suppression of neuroprotective irisin [61], which may mechanistically explain why the sarcopenic obesity phenotype carries greater dementia risk than either condition alone, highlighting the importance of maintaining both metabolic health and muscle integrity for cognitive preservation.

This study provides, to our knowledge, the first large-scale, nationally representative investigation of SO prevalence, influencing factors, and dementia association in China, addressing critical gaps in Asian epidemiology. Methodological rigor was demonstrated through a sophisticated Bayesian CAR model that generated precise, spatially adjusted prevalence estimates revealing a significant north-south gradient. The combined use of traditional multivariable logistic regression and XGBoost modeling provided additional insights by quantifying the relative contributions of identified influencing factors. The adoption of regionally appropriate diagnostic criteria (AWGS 2019), robustness confirmed through sensitivity analyses, and inclusion of relevant covariates strengthen the findings. Notably, this study provides novel clinical evidence for the SO-dementia association in an Asian population using a temporal design, complementing previous Western-focused research. However, this study has several limitations that warrant consideration. First, regarding study design, the cross-sectional analysis of factors associated with SO precludes causal inference, indicating only statistical associations. Although the temporal separation between SO assessment and dementia diagnosis strengthens the longitudinal analysis, the single-timepoint measurement of SO prevents an examination of its dynamic effects on dementia risk. Second, methodologically, the use of operational definitions for sarcopenia and obesity that lack a global consensus may affect the comparability of our findings. Third, despite extensive covariate adjustment, residual confounding from unmeasured variables (e.g., socioeconomic status, detailed lifestyle behaviors) remains possible, necessitating cautious interpretation of any causal claims. Finally, potential selection bias due to the exclusion of participants (mostly because of missing dementia assessments) and unaddressed mortality as a competing risk are limitations of this study. However, the largely similar baseline characteristics between the included and excluded cohorts (Table S5) suggest that such bias is unlikely to have substantially altered the main findings. The results should therefore be interpreted with this context in mind.

## Conclusion

Our study identifies significant geospatial disparities in SO prevalence and influencing factor effects across China’s seven geographic regions. Older age, female sex, hypertension, and more severe depressive symptoms were significantly associated with higher SO prevalence. Notably, individuals with SO exhibited increased dementia risk, underscoring the urgent need for targeted interventions in China to better manage sarcopenia and obesity, thereby mitigating SO’s detrimental cognitive effects.

## Supplementary Information

Below is the link to the electronic supplementary material.

Supplementary file1 (DOCX 830 KB)

## Data Availability

The CHARLS datasets for this study are publicly available and can be found here: [http://charls.pku.edu.cn/.](http:/charls.pku.edu.cn).
