# Supplementary material for: Prevalence, influencing factors, and dementia outcome of sarcopenic obesity in China
Source: Aging Clin Exp Res. 2026 Jan 13;38(1):49. doi: 10.1007/s40520-025-03318-8 (PMC12819534; doi:10.1007/s40520-025-03318-8)
Supplement: Supplementary file 1 — Supplementary file1 (DOCX 830 KB) [file 40520_2025_3318_MOESM1_ESM.docx]

**Prevalence, influencing factors, and dementia outcome of sarcopenic obesity in China**

Excluded participants with missing data on basic characteristics and other relevant variables (N=9,457)

10,256 participants for prevalence and risk factor analysis of SO

Excluded participants who lacked a dementia survey in 2015 or 2018, or who had a dementia diagnosis in 2015 (N=4,558)

A total of 21,095 participants were recruited in the CHARLS 2015

5,698 participants for SO and dementia incidence risk analysis

9,601 participants without SO

655 participants with SO

Excluded participants under 45 years old (N=1,382)

5,330 participants without SO

368 participants with SO

**Figure S1** The selection process of this study participants.

**Table S1** List of geographical region divisions in mainland China

| Geographical region | Include provinces and cities |
| --- | --- |
| Northeast | Heilongjiang, Jilin, Liaoning |
| North | Inner Mongolia, Beijing, Tianjin, Hebei, Shanxi |
| Central | Henan, Hubei, Hunan |
| East | Shandong, Jiangsu, Anhui, Shanghai, Zhejiang, Jiangxi, Fujian |
| South | Guangxi, Guangdong |
| Northwest | Xinjiang, Qinghai, Gansu, Shaanxi |
| Southwest | Sichuan, Chongqing, Yunnan, Guizhou |

**Definition and Ascertainment of Sarcopenia**

Sarcopenia was defined according to the 2019 Asian Working Group for Sarcopenia (AWGS 2019) criteria, which are based on three core components: low muscle strength, low muscle mass, and low physical performance [1]. As the CHARLS 2015 survey did not include direct measurements of appendicular skeletal muscle mass (ASM) by dual-energy X-ray absorptiometry (DXA) or bioelectrical impedance analysis (BIA), we employed a modified operationalization of these criteria [2,3]. The specific assessment methods for each component were as follows:

**(1) Muscle Strength**
Handgrip strength, assessed with a dynamometer, was used to evaluate muscle strength. Low muscle strength was defined using the AWGS-recommended cut-offs of <28 kg for men and <18 kg for women. The measurement protocol required participants to stand upright with the elbow flexed at 90°. After the investigator ensured the dynamometer was correctly adjusted, participants were instructed to squeeze it with maximum effort for several seconds. Each hand was tested twice with adequate rest between trials. The average value for each hand was calculated, and the higher of the two averages was used in the analysis. To ensure safety and data accuracy, individuals who had undergone hand surgery or reported significant hand pain, swelling, inflammation, or injury in the preceding six months were excluded from this assessment.

**(2) Muscle Mass**
Given the lack of direct ASM measurements, low muscle mass was estimated using the validated appendicular skeletal muscle mass index (ASM/Ht²). This proxy has demonstrated high concordance with DXA measurements in Chinese populations [4]. The index was calculated using the following equation:
ASM/Ht² = [0.193 × weight (kg) + 0.107 × height (cm) - 4.157 × sex (male=1; female=2) - 0.037 × age (years) - 2.631] / [height (m)]².
Sex-specific cut-offs of ASM/Ht² < 7.00 kg/m² for men and < 5.28 kg/m² for women were applied to define low muscle mass.

**(3) Physical Performance**
Physical performance was assessed using the five-time chair stand test. A completion time of ≥12 seconds was considered to indicate low physical performance, consistent with the AWGS 2019 guidelines.

A participant was diagnosed with sarcopenia if they met the criterion for low muscle mass plus either low muscle strength or low physical performance.

**References**

[1] Chen LK, Woo J, Assantachai P, et al. Asian Working Group for Sarcopenia: 2019 Consensus Update on Sarcopenia Diagnosis and Treatment. *J Am Med Dir Assoc*. 2020;21(3):300-307.e2.

[2] Hu Y, Peng W, Ren R, Wang Y, Wang G. Sarcopenia and mild cognitive impairment among elderly adults: The first longitudinal evidence from CHARLS. *J Cachexia Sarcopenia Muscle*. 2022;13(6):2944-2952.

[3] Kitamura A, Seino S, Abe T, et al. Sarcopenia: prevalence, associated factors, and the risk of mortality and disability in Japanese older adults. *J Cachexia Sarcopenia Muscle*. 2021;12(1):30-38.

[4] Wu X, Li X, Xu M, Zhang Z, He L, Li Y. Sarcopenia prevalence and associated factors among older Chinese population: Findings from the China Health and Retirement Longitudinal Study. *PLoS One*. 2021;16(3):e0247617. Published 2021 Mar 4.

**Table S2** Variables for the CHARLS 2015 study participants.

| **Variables** | **Definition** | **Code** |
| --- | --- | --- |
| Gender | Gender of the respondents | 0: Male; 1: Female |
| Marital Status | Marital Status the respondents | 0: Married; 1: Unmarried |
| Residence | The household registration of respondents | 0: Urban; 1: Rural |
| Drinking Status | the frequency of respondents drinking alcohol in the past year | 0: Never drinking; 1: Have drunk alcohol at present or before |
| Hypertension | Respondents are diagnosed of hypertension | 0: No; 1: Yes |
| Diabetes | Respondents are diagnosed of diabetes | 0: No; 1: Yes |
| Depressive status | Respondents completed a score of ≥10 on the Center for Epidemiological Studies Depression Scale | 0: No; 1: Yes |
| Social Participation | Respondents did not engage in any of the following social activities in the past month:  1. Interacted with friends;  2. Played Ma-jong, played chess, played cards, or went to community club;  3. Provided help to family, friends, or neighbors who do not live with you  4. Went to a sport, social, or other kind of club;  5. Took part in a community-related organization;  6. Done voluntary or charity work;  7. Cared for a sick or disabled adult who does not live with you  8. Attended an educational or training course  9. Stock investment  10. Used the Internet | 0: No; 1: Yes |

**
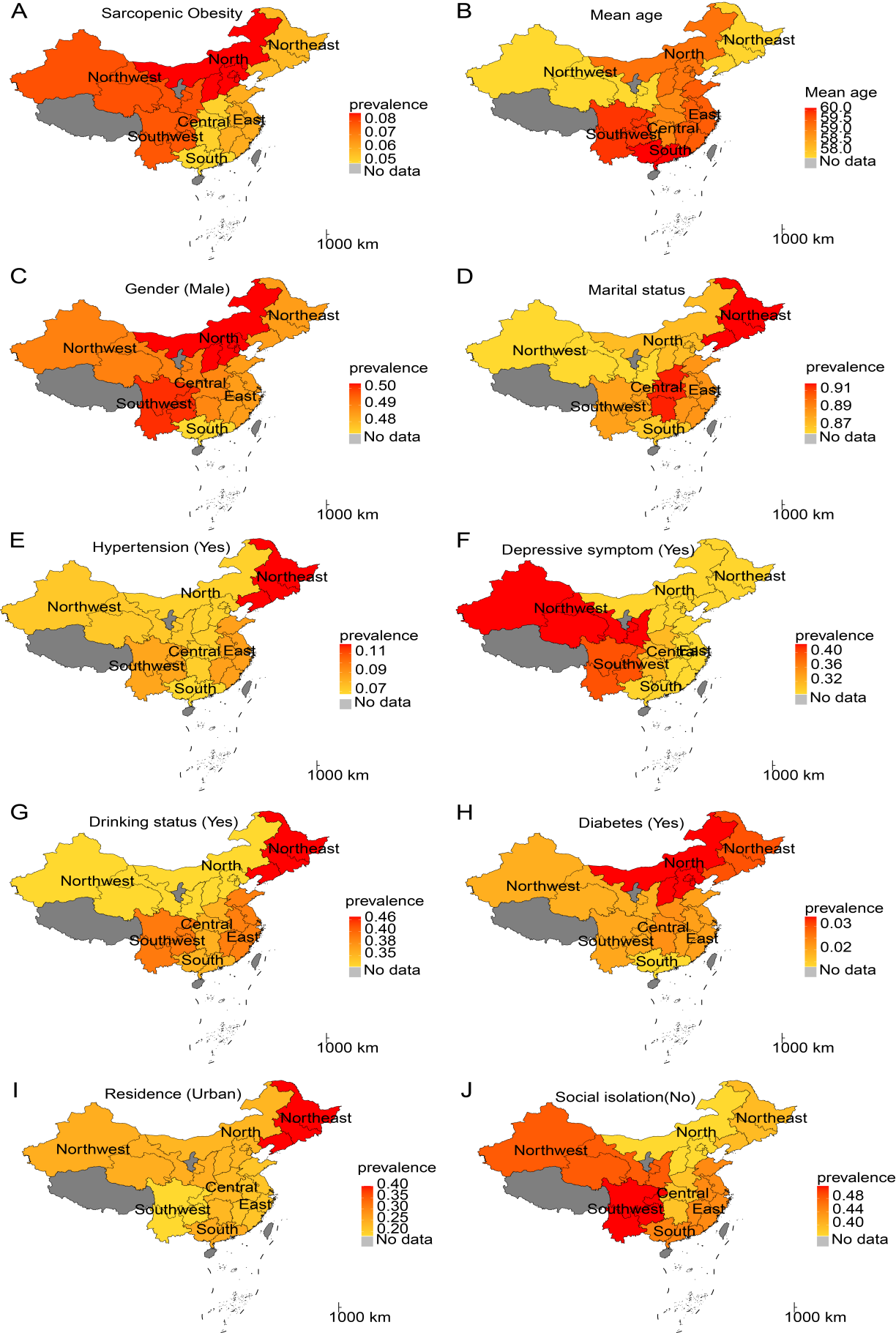
Figure S2** Prevalence of SO and related covariates across China. (A) SO. (B) Age. (C) Gender. (D) Marital status. (E) Hypertension. (F) Depressive symptoms. (G) Drinking status. (H) Diabetes. (I) Residence. (J) Social isolation.


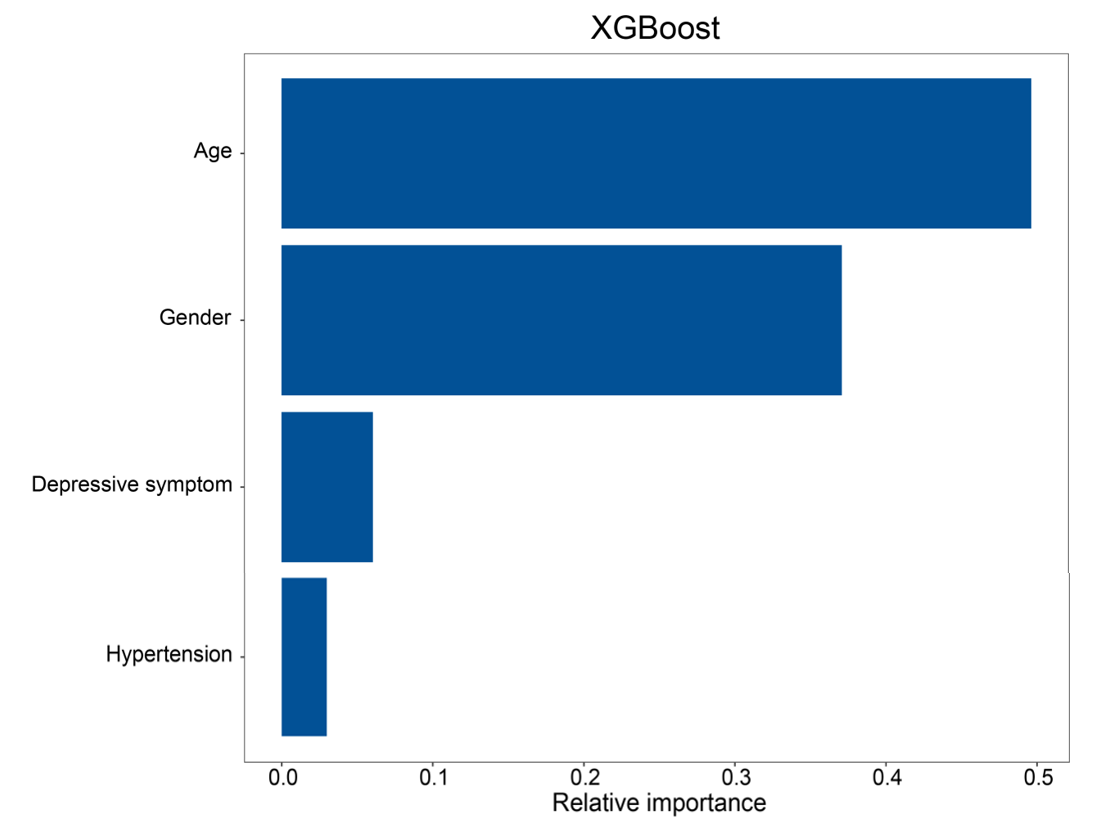


**Figure S3** Relative importance of risk factors of SO.

**Table S3** Region-specific OR of SO risk factors

| Region | Age | *P value* | Gender | *P value* | Depressive symptom | *P value* | Hypertension | *P value* |
| --- | --- | --- | --- | --- | --- | --- | --- | --- |
| North | 1.11  (1.07-1.15) | <0.001 | 3.95  (2.06-7.99) | <0.001 | 1.38  (0.76-2.46) | 0.287 | 4.42 (1.88-10.00) | <0.001 |
| Northeast | 1.09  (1.06-1.12) | <0.001 | 3.88  (1.94-8.23) | <0.001 | 1.12  (0.63-1.94) | 0.697 | 1.03 (0.43-2.22) | 0.939 |
| East | 1.09  (1.07-1.12) | <0.001 | 5.76  (3.83-8.89) | <0.001 | 1.86  (1.36-2.55) | <0.001 | 1.24 (0.75-1.97) | 0.386 |
| Central | 1.11  (1.08-1.14) | <0.001 | 4.14  (2.29-7.85) | <0.001 | 1.73  (1.06-2.80) | 0.027 | 1.21 (0.49-2.59) | 0.659 |
| South | 1.08  (1.04-1.12) | <0.001 | 4.74  (2.09-12.30) | <0.001 | 1.08  (0.54-2.11) | 0.823 | 2.43 (0.83-6.22) | 0.079 |
| Southwest | 1.09  (1.07-1.11) | <0.001 | 5.18  (3.30-8.36) | <0.001 | 1.32  (0.91-1.91) | 0.138 | 1.72 (0.98-2.92) | 0.050 |
| Northwest | 1.10  (1.06-1.14) | <0.001 | 8.84  (4.00-22.03) | <0.001 | 1.08  (0.61-1.93) | 0.793 | 2.86  (1.17-6.52) | 0.015 |

Notes: OR, odds ratio; SO, sarcopenia obesity.


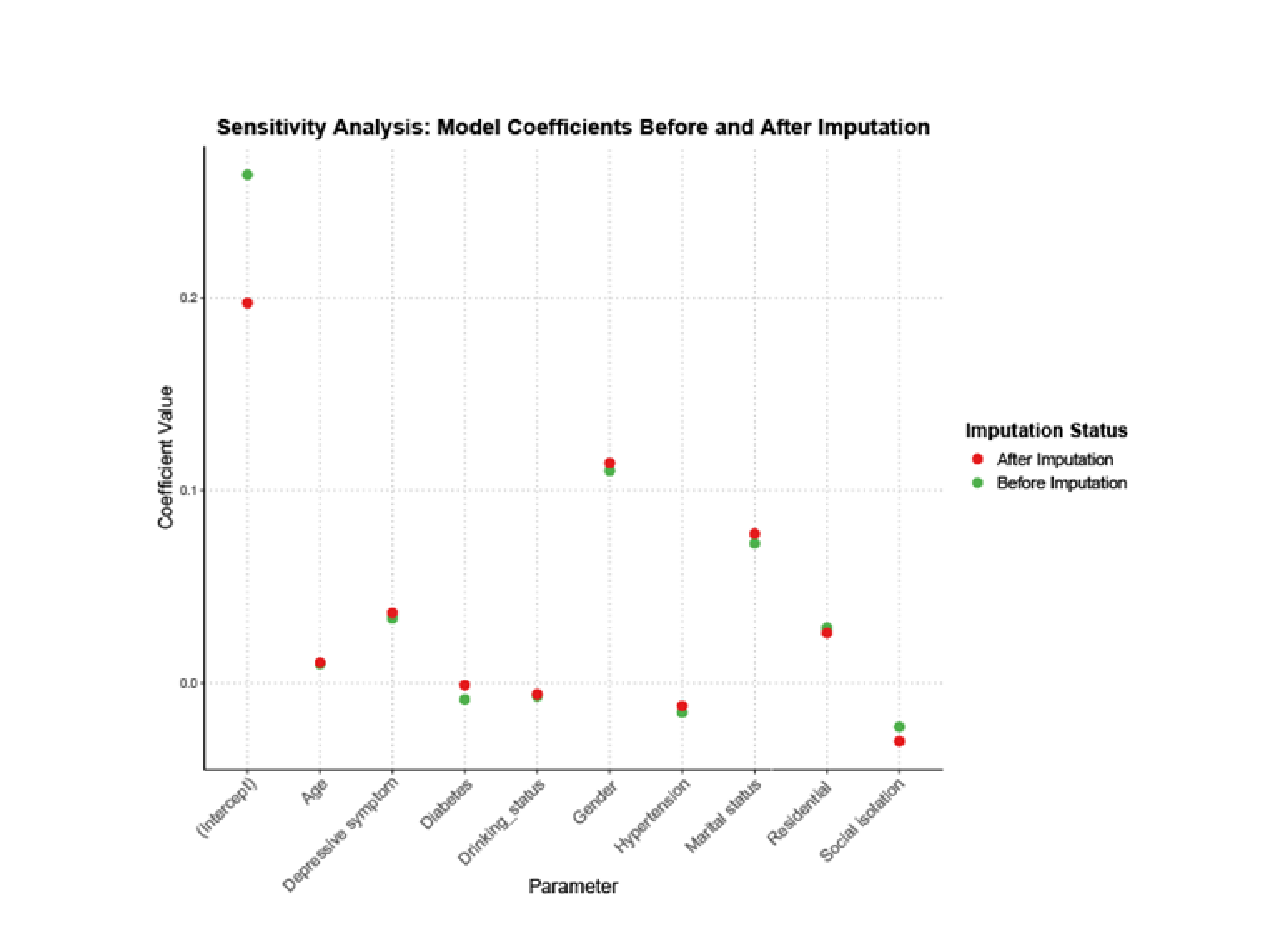


**Figure S4** Sensitivity analysis was based on multiply imputed data.

Note: Five imputed datasets were generated using the Multivariate Imputation by Chained Equations (MICE) approach, and the final parameter estimates were obtained by pooling results across these datasets using Rubin's rules.

**Table S4** Associations between SO with dementia and TICS-based global cognitive scores based on multiply imputed datasets

| **Outcome** | **Effect values** | **-** |
| --- | --- | --- |
| **Dementia** | **OR (95% CI)** | ***p* value** |
| Group 1 | 1.00 (Reference) | - |
| Group 2 | 1.17 (0.85, 1.61) | 0.330 |
| Group 3 | 1.82 (1.65, 2.01) | <0.001 |
| Group 4 | 1.90 (1.51, 2.38) | <0.001 |
| *p* for trend | <0.001 | - |
| **TICS-based global cognitive scores** | **β (95% CI)** | ***p* value** |
| Group 1 | 1.00 (Reference) | - |
| Group 2 | -0.84 (-1.64, -0.04) | 0.040 |
| Group 3 | -1.31 (-1.89, -0.74) | <0.001 |
| Group 4 | -1.42 (-2.54, -0.30) | 0.019 |
| *p* for trend | <0.001 | - |

Notes: SO, sarcopenic obesity; TICS, Telephone Interview for Cognitive Status; OR, odds ratio; CI, confidence interval; β, beta; model was adjusted for age, gender, marital status, hypertension, diabetes, depression status, residence, alcohol consumption, and social participation. Group 1: normal weight and without sarcopenia; Group 2: obesity and without sarcopenia; Group 3: normal weight and with sarcopenia; Group 4: SO.

**Table S5** Comparison of baseline characteristics between the included population and the excluded population in the SO-dementia association analysis

| Characteristics | Included  (N = 5698) | Excluded  (N = 4558) | *P* value |
| --- | --- | --- | --- |
| Age (year), mean (SD) | 60.24 (8.27) | 61.08 (8.51) | 0.108 |
| Gender, n (%) |  |  |  |
| Male | 2741 (48.10) | 2187 (47.98) | 0.604 |
| Female | 2957 (51.90) | 2371 (52.02) |  |
| Marital status, n (%) |  |  |  |
| Married | 4787 (84.01) | 4086 (89.65) | <0.001 |
| Unmarried | 911 (15.99) | 472 (10.35) |  |
| Hypertension, n (%) |  |  |  |
| Yes | 2026 (35.56) | 1661 (36.44) | 0.274 |
| No | 3672 (64.44) | 2897 (63.56) |  |
| Depressive symptoms, n (%) |  |  |  |
| No | 3831 (67.24) | 3021 (66.27) | 0.179 |
| Yes | 1867 (32.76) | 1537 (33.73) |  |
| Drinking status, n (%) |  |  |  |
| Yes | 2199 (38.60) | 1635 (35.86) | 0.042 |
| No | 3499 (61.40) | 2923 (64.14) |  |
| Diabetes, n (%) |  |  |  |
| Yes | 782 (13.73) | 567 (12.43) | 0.053 |
| No | 4916 (86.27) | 3991 (87.57) |  |
| Residence, n (%) |  |  |  |
| Urban | 3503 (61.47) | 2679 (58.77) | <0.001 |
| Rural | 2195 (38.53) | 1879 (41.23) |  |
| Social participation, n (%) |  |  |  |
| No | 3669 (64.39) | 2859 (62.73) | 0.141 |
| Yes | 2029 (35.61) | 1699 (37.27) |  |
| SO, n (%) |  |  |  |
| No | 5330 (93.54) | 4281 (93.92) | 0.507 |
| Yes | 368 (6.46) | 277 (6.08) |  |

*Notes:* SD, standard deviation; SO, sarcopenia obesity.
